# Beyond Tolerance: Mitigating Human–Wildlife Conflict with Hospitality

**DOI:** 10.3390/ani14081185

**Published:** 2024-04-15

**Authors:** Christopher Serenari

**Affiliations:** Department of Biology, Texas State University, San Marcos, TX 78666, USA; c_s754@txstate.edu

**Keywords:** hospitality, human–wildlife conflict, tolerance, wildlife

## Abstract

**Simple Summary:**

In search of an alternative standard for an increasingly divided world there has been a rise in scholarly interest in non-commodified hospitality to achieve sustainable human–human and human–Nature relations. Unlike tolerance, hospitality offers us the intellectual space required to rethink human–wildlife relations in a way that reverses the anthropocentric power dynamic undergirding tolerance and creates hospitable spaces for wild animals on a crowded planet. This conceptual scoping project engages a thorough critique of tolerance as a design principle within wildlife conservation governance, particularly human–wildlife conflict, and proposes a more durable human–wildlife coexistence arrangement underpinned by hospitality.

**Abstract:**

Tolerance has become a central position in wildlife conservation thought, and a goal in and of itself. Appeals to tolerance are expected to grow as the planet becomes more crowded, species are lost, and habitat is degraded. The concept has been uncritically adopted in wildlife conservation to mitigate human–wildlife conflicts (HWCs). However, scholars have demonstrated that tolerance is burdened with limitations, paradoxes, and shortcomings. Thus, blind adherence to it is not expected to produce a coexistence design necessary to sustain wildlife populations in the long term. This paper is a conceptual scoping project that engages a summary and critique of tolerance as a design principle within wildlife conservation governance. After introducing a resultant theory of dysfunctional human–wildlife coexistence, a pathway toward hospitality as a social institution is outlined via several commitments societies can make to transition to an era of normalizing a process of sincere welcoming, care, and support. The transition from tolerance to hospitality will entail shifting responsibility to humans to modify their behavior to help keep wildlife invisible where it is essential, learning about what wildlife want and need, and ensuring wildlife is not injured for being themselves.

## 1. Introduction

Human–wildlife conflict (HWC, negative interactions between humans and wildlife or between humans about wildlife) is a complex global problem, often resulting in notable and unsustainable losses for humans and wildlife [1,2]. Despite vast resources being deployed to mitigate HWC, it often endures in a perpetual state of non-resolution, which can produce or exacerbate negative views and behaviors toward wildlife [3]. Some researchers have indicated that intervention efforts may make little to no substantive impact on the frequency, intensity, or perpetual nature of HWC [4,5]. The most common solution for HWC is often for humans to tolerate or bear the burden of wildlife, either to facilitate the propagation of a species or to encourage a cohabitation arrangement between humans and wildlife [6].

Emerging from a belief that it is critical for overcoming racial, religious, and ethnic tensions in liberal democracies [7], tolerance has become a central position in wildlife conservation thought, deemed vital to human–wildlife coexistence (e.g., space sharing) arrangements [8,9] as well as a conservation goal in and of itself [10]. Thus, appeals to tolerance are expected to grow as the planet becomes more crowded, species are lost, and habitat is degraded [10]. Evidence of tolerance’s institutional embedding as a modus vivendi in the United States has been revealed by, for instance, the U.S. Fish and Wildlife Service director stating that tolerance is elemental to extinguishing HWC where conservation occurs in human-dominated landscapes [11] or non-governmental wildlife organizations arguing that ranchers’ intolerance is the root cause of their resistance to bison in Montana [12]. 

Alternatively, insights from critical studies suggest that the potential for tolerance to mitigate HWC is limited. Tolerance is an important concept with universal principles to resolve political and cultural conflicts [13] and has permeated and shaped conservation ideology in theory and practice. Yet, it is multi-layered with political undercurrents, plagued by paradoxes, and operationalized through its contextualization, rendering its universal efficacy limited and, thus, not always helpful, harmful, or as advertised [14]. In this essay, the contention is made that a historical tolerance paradigm in the domain of wildlife conservation is unsatisfactory for solving HWC and achieving viable coexistence arrangements in the long term. In its place, conservation hospitality provides a more realistic alternative to address these shortcomings of tolerance, encourage long-term thinking, and bring humans and wildlife closer together in these challenging times to achieve a more civilized planet. 

The purpose of this conceptual scoping project is twofold. One aim is to engage a critique of tolerance as a design principle [15] within wildlife conservation governance. Design principles form the boundaries of, in this instance, human–wildlife interactions to define and direct how coexistence is facilitated. The second aim is to advocate for a durable human–wildlife coexistence arrangement underpinned by hospitality. 

## 2. A Summary of Tolerance

Though tolerance was deliberated over by prominent ancient philosophers debating how to confront the deviant and inappropriate, its status as a Western virtue emerged in Locke’s 17th century reflections on engaging religious difference. Explorations and debates about tolerance concentrate on human–human relations, particularly how humans react when they confront areas of disagreement—hence, an objection component is elemental [16]. Considering tolerance as a process, Conway [17] (p. 2) wrote:

Broadly conceived, tolerance involves choosing wisely in relation to the fact of diversity, specifically the encountering of differences viewed as morally problematic, distasteful, disagreeable or erroneous…experiences of difference that evoke a response of being ill-at-ease, suspicious, and distrustful which can lead to reactions of alienation, dread, resentment, and hostility.

In liberal democracies, tolerance is also considered the voluntary practice of toleration within a framework of equality [18,19]. Forst asserted that tolerance is a voluntary personal ethic, neither virtue nor value, and arising from an ethical, belief-based (not moral) objection to difference (via enduring or total rejection) [14]. It is balanced by an acceptance component where there is an understanding that individuals differ in tastes, lifestyles, and politics, but they have a Kantian right to exist in their difference. 

According to Forst [20], four contemporary conceptualizations of tolerance exist, each designed and bolstered by a unique constellation of socio-political norms. The first, labeled *permission*, embodies a classic hierarchical/vertical power dynamic where a different minority is permitted by the dominant group or authority to live as they desire, within limits imposed by the group in power. The second is considered a *coexistence* conception, where the differing parties share power horizontally, agreeing to a peaceful arrangement unless the power balance shifts. A third is characterized as a *respect* conceptualization. This form of tolerance necessitates that all parties develop norms to recognize their differences and refrain from contesting the other’s socio-political framework. The final conceptualization is *esteem.* In this more socially demanding form of tolerance, ethical reverence is required to operationalize mutual recognition where an alternative way of life is viewed as noble and justified though not as ideal as one’s own. 

Incorporating discourse analysis, Brown [7] (p. 4) characterized tolerance as a historically and politically grounded discursive process targeting the regulation of the objects of tolerance, such as lifestyles, races, and regimes, constituting territories and identities. Her critique of tolerance discourse elucidates operations of power, histories, cultures, governance, and subject production. Tolerance “produces and positions subjects, orchestrates meanings and practices of identity, marks bodies, and conditions political subjectivities” through the production and dissemination of its discourse throughout societies. Thus, tolerance is not a benign virtue that emerges from the objection to another’s beliefs. Rather, Brown noted that contemporary tolerance talk is inherently power-infused and political, aimed at people, things, practices, and cultures. Emerging from settler–native encounters, tolerance discourse reached a zenith in the 20th century as Western societies tried to address racial and religious differences and enhance justice and peace. Tolerance discourse is universal, having been applied to a range of topics by liberals, conservatives, government institutions, religious groups, and others. It is Brown’s tolerance as discourse that provides intellectual space to consider how conceptualizations of tolerance should not be uncritically promoted.

Though definitions and operationalizations vary, social psychology studies within wildlife social science, in particular, have communicated the concept’s viability as a research trajectory [21]. Often considered a behavioral or cognitive state [22,23], social science has inconsistently characterized and measured the term, with early investigations settling on the tolerance–intolerance binary, while recent attempts have been made to clarify its framing, operationalization, and measurement by joining attitude, normative beliefs, and behavioral intention [8]. Psychological models attempt to predict, for instance, the role of perceived costs and benefits and cognitive dimensions, such as attitudes and beliefs, in motivating one’s capability to share space with wildlife and determine under what conditions humans would do so [6,23,24]. Hence, models are inherently anthropocentric and, thus, a hierarchical power structure is evident.

As a popular baseline for managing human–wildlife interactions, a tolerance paradigm requires societies to commit to a particular version of coexistence where unfavorable wildlife qualities (i.e., a threat to property or human safety) compel human thought and behavior—as if we are waiting for a negative outcome to occur. Tolerance is underscored by and induces judgements about acceptable risks [25], compliance with social norms (e.g., adhering to laws), or feelings of powerlessness that precede the act of toleration [26]. When tolerance is deemed deficient, coexistence arrangements can be considered thin [27], dysfunctional, incomplete [28], or negative [29]. If tolerance is vital to human–wildlife coexistence arrangements, where dislike of the unfavorable or unfamiliar is an inherent attribute, we must clarify the durability of tolerance to serve as a long-range coexistence design principle. The next section summarizes critiques of tolerance to draw attention to problems with blind adherence to it, with a focus on addressing HWC. 

### 2.1. A Critique of Tolerance

#### 2.1.1. Problem 1: Limited Conceptualization in Wildlife Contexts

In wildlife contexts, tolerance is assessed through a psychological lens, privileging individual-level cognitions and behaviors [30]. It is commonly commingled with acceptance and is assumed to connote positive thinking [10]. Outside of wildlife social science, however, tolerance has been reasoned to be an incoherent, inconsistent, and problematical concept. For example, Neges [31] noted that the term can materialize as affirmation of difference (e.g., another’s opinion), indifference, or condemnation as a function of powerlessness to change an outcome. Another critique is that the space for tolerance in practice is limited or afflicted by contradictions and tensions [32,33]. Esteva [34] and other scholars assert that to tolerate is merely another form of intolerance, which breeds social, cultural, and political instability within a community or society [17]. The paradox of the tolerant racist embodies such incongruity whereby the remedy for racism is that the racist merely needs to become more tolerant [35]. Ricoeur [36] declared that tolerance can only exist after intolerance has been defeated, but that accomplishment is unlikely in an age of cultural relativism where everyone is right or legitimated [37] and intrinsic intolerance structurally and culturally persists (e.g., select religions, economic thought). Moreover, tolerance is a Western construct and not universally practiced [38]. These and other powerful characterizations and critiques of tolerance have largely been overlooked by wildlife social science thus far, though they are critical for determining the viability of the concept as a conservation project and design principle. 

#### 2.1.2. Problem 2: Arbitrary Limits to Wildlife’s Existence

The classic conceptualization of tolerance in HWC contexts suggests a hierarchical design and, therefore, aligns with a *permission* conceptualization, where humans dictate the processes of objection, acceptance, and rejection. It is the least costly form of tolerance in terms of (a) the need for the dominating group to adapt or change and (b) negative outcomes experienced by the dominated. The notion of drawing limits to another’s existence emerged from Locke’s [39] account of religious tolerance. In his view, to tolerate is to refuse suppression, persecution, and denial. The more powerful actor could but does not interfere with the life of the other and permits their existence so long as there is no need to interfere. Because tolerance does not account for our inherent connections to Nature [40], the notion of tolerance in HWC aligns with the observation that tolerance resembles what Habermas et al. [41] characterized as a weak anthropocentric and charitable position. Humans, as the more powerful actor and without a way to verbally communicate with animals, arbitrarily set the levels of deviation from some acceptable norm or disfavor for select wildlife behavior (i.e., the reason of the strongest, [42]). Problematically, however, “the strongest” can suspend their engagement in the act of toleration of wildlife at almost any time, which also renders tolerance transitory. Thus, what we observe in practice is an overemphasis on the individual performance of subjective right or good acts, particularly those that are voluntaristic and self-serving (i.e., anthropocentric environmental stewardship, [43]). Wildlife conservationists and advocates can merely cross their fingers that these actors refrain from exercising the power they have over animals. It is this static power dynamic that fuels anthropocentrism, which underpins tolerance and is a primary cause of species loss [44] and HWC [45]. Thus, without a societal movement to redefine humanism using an ecological framing [46], it is doubtful that humans can continue to exercise a permission form of tolerance of wildlife and expect to reverse these trends.

#### 2.1.3. Problem 3: Denial of the Animal Other

Because acts of tolerance do not confront humans’ difficulty reconciling the animal other, scholars argue tolerance connotes a perpetual state of non-recognition or denial of non-human animals [47,48]. Those in power have the power to exclude, which is essential to the exercise of othering, a form of oppression [49]. Othering helps humans stand in contrast to non-human animals, render our similarity to other humans distinct, and distance ourselves from animals on at least moral, ethical [50], or utility [51] grounds. For millennia, the challenge posed by the otherness of wildlife has produced an array of human responses. Human representations of wildlife and human–wildlife interactions are based on deeply embedded socio-cultural conditions from which one’s worldview originates (e.g., animals are machines [Descartes], animals are not rational or self-aware [Kant], animals deserve humane treatment [Bentham] [52]). In many global contexts, codification of those views and experiences into rules or laws occurs [53]. A primary thrust of perpetual non-resolution to HWC is negation of wildlife’s need to occupy ecological niches where human settlements occur [54]. Humans’ inability to address the animal other in this regard has been deemed an ethical trap [54] that yields speciesism and human exceptionalism [50], which then produce violent outcomes that undermine efforts to develop relational approaches needed to address HWC across extended spatial and temporal scales. These critiques compel us to confront how acts of tolerance and intolerance in HWC settings are merely ineffective attempts to master a human–animal dynamic that simply cannot be conquered—the impossible—via exclusionary thinking [48].

#### 2.1.4. Problem 4: A Perpetual State of Negative Peace

Recent research has highlighted that coexistence comprises an intolerance–tolerance dichotomy and attempts to situate tolerance in terms of negative–positive thinking and behavior. For instance, Bhatia et al.’s [29] conceptualization of negative–positive coexistence is worthy of mentioning because it captures a gradient situated within the intersection of weak–strong and negative–positive peace (i.e., intolerance [i.e., negative attitudes and behavior] with stewardship [i.e., positive attitudes and behavior]). However, Pasamonk [38] outlined that the assumption that tolerance is intrinsically positive is problematic because the limits of tolerance are subjective and contextual, and conflicts are often a result not of divergent attitudes but truths. In some cases, there is no room for an alternate truth or unconditional tolerance (e.g., moral conflict), and toleration of the intolerant makes little rational sense. Tolerance emerged “in response to exhaustion from suffering caused by intolerance…the realities of the contemporary world call us to the challenges of a far more positive virtue” [17] (pp. 2, 8) but resulted in an entrenched state of “resigned acceptance of lamentable difference for the sake of peace”. Thus, rejection and denial are at the heart of tolerance but go unaddressed by a conceptualization of tolerance that assumes the universality of political, ethical, or epistemological truths [38]. To elaborate on how rejection of denial needs to be a central aspect of toleration, I turn to functional coexistence theory (FCT) to make the point that the best we are producing with appeals to tolerance in HWC settings is not positive but negative thinking and manifests a dysfunctional form of coexistence.

Scholars of FCT have captured interactions between human actors and characterized relations as intentionally limited and non-violent, fueling a perpetual state of non-resolution to conflict [55]. Functional coexistence in the context of human–human interactions includes a range of coexistence types, arranged in terms of the degree to which adversaries are recognized and legitimated [56]. Power may be shared or asymmetrical but exercised to create and defend conditions that preserve a state of negative peace between adversaries. 

**Figure 1:**
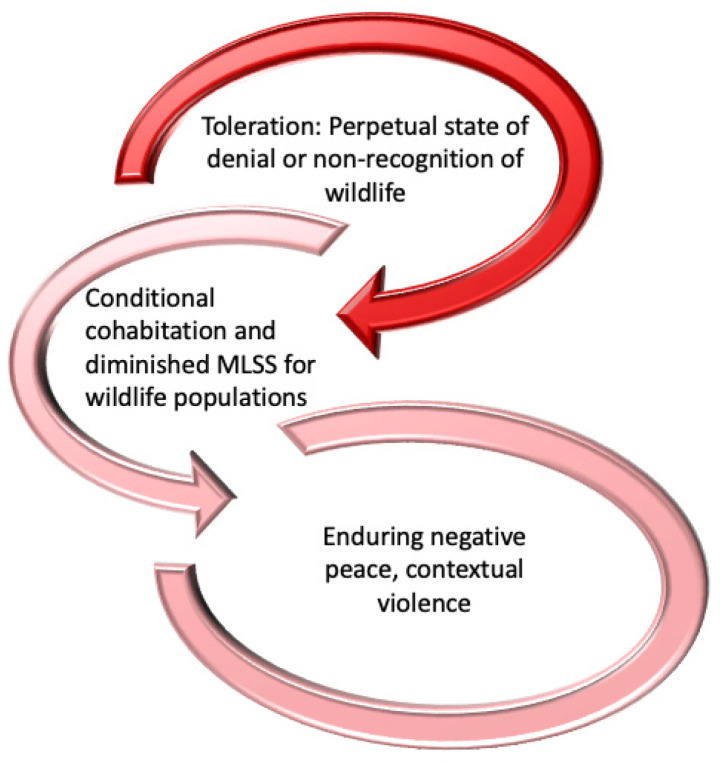
Theory of dysfunctional human–wildlife coexistence.

Applying FCT to human–wildlife relations is an innovative and, arguably, necessary adaptation of the concept because perpetual states of anthropocentrism, negativity, and contextual violence (material or silent, [e.g., lethal removal or structurally sanctioning it]) within HWC contexts exist (Figure 1). In practice, forms of functional human–wildlife coexistence that primarily serve human interests have been established through, for example, the denial of a species’ right to exist (e.g., extirpation), recognition via acquiescence or apathy (forms of tolerance), and mutually beneficial relations (e.g., wildlife watching, acts some call stewardship [e.g., creating habitat]). The minimum livable social space (MLSS) is a key concept within FCT but is missing from coexistence conceptualizations, especially ones that conflate tolerance, coexistence, and stewardship or their attributes (the reader can identify these instances for themselves). The MLSS comprises the context by which each party involved in conflict is provided its minimum needs to ensure long-term survival, health, and, often neglected, dignity. In applying FCT to HWC contexts, we observe that humans have difficulty (a) ceding control over human–wildlife interactions and their outcomes, thereby producing chronic deferral to anthropocentric approaches to mitigate HWC and (b) agreeing on an MLSS for all species involved. Despite recent attempts by researchers to enfold tolerance within a “positive” coexistence arrangement (“social justice, well-being, and harmony for all”, [57] (p. 1)), FCT theory suggests that systemic and sustained human disallowance of an MLSS for wildlife in defense of contextual violence will never allow a positive anthropocentric coexistence arrangement to permeate societies and ecosystems. Rather, adapting Esteva’s [34] view, the current spirit of tolerating the animal other is to declare, *You are not what I want or need you to be, nor are like me, so I will tolerate you if I cannot kill, block, or remove you from my MLSS*. Researchers acknowledge that conflict is inherent to human coexistence with wildlife [58], but such a view does not confront Goethe’s [59] (p. 507) argument that “to tolerate is to insult” and, thus, negation of the animal other persists (see Problem 3). 

From this analysis arises the concern that we do not fully understand how perpetual states of non-recognition of the animal other and negative peace (via intolerance and tolerance) affect our ability to relate to or connect with wildlife to meet the challenges set forth by the human-caused Sixth Mass Extinction crisis. Moreover, though animals cannot communicate the implications of being the object of toleration, research demonstrates they are often negative for humans targeted for toleration [60]. Hence, it seems unlikely that tolerance can produce the ideal type of stewardship needed to foster closer relations between humans and Nature. Instead, our reliance on tolerance will continue to manifest not as a form of mutuality or recognition of our interdependence on Nature, but as the continued denial of an MLSS for wildlife.

#### 2.1.5. Problem 5: Depoliticizing Human–Wildlife Encounters

Tolerance is a political problem that has been depoliticized to make it easier for the powerful to portray toleration as a benign, innocent, or cognitive socio-political process of suppressing full and equal rights for any entity subjected to it [61]. A political vocabulary is required to reveal how invoking tolerance limits the conceiving and viability of alternative actions and avoids debates about priorities, biases, truth, and ethics. Discussing the problem of tolerance in liberal democracies, Brown [7] articulated how an apolitical tolerance discourse eliminates our ability to acknowledge and confront its historical and power-infused origins and, instead, frames tolerance as a therapeutic or behavioral project instead of a justice project (p. 16). 

Applying Brown’s [7] (p. 15) sentiment to the HWC context reveals that tolerance discourse reduces HWC to an “inherent friction” between species and makes differences between humans and wildlife a source of conflict, one that calls for the practice of tolerance. However, an apolitical tolerance overlooks how taught tolerance emerges, replicates, and affects how communities negotiate one’s difference with wildlife, how hegemonic norms justify forms of violence, or what strategies and tactics managers use to address HWC. Findings from McKiernan and Instone [62] demonstrated that HWC discourse is a powerful shaper of human–wildlife relations, resulting in the negation of the avian animal other (Problem 3). Further, an apolitical tolerance discourse masks deeper systemic problems that prevent humans’ ability to thoughtfully govern HWC in ways that break away from a paradigm promoting improved manners (i.e., appeals to stewardship) and attending to the idea that if animals could, they would indeed resist the existing anthropocentric and hierarchal arrangement. Thus, tolerance discourse is a technocratic fix that does, under certain conditions, exacerbate the very problem its deployment is meant to resolve while formidable drivers of HWC, such as political economic structures [63], are downplayed. In contemporary political discourse about HWC, wildlife is still forced to conform to the unsustainable world humans have created.

## 3. Where Do We Go from Here?

Wildlife is powerless to be any different from humans nor can they negotiate, agree, or collaborate to develop terms of coexistence. Our inability to communicate with the animal other means that adoption of a code of mutual tolerance to promote peaceful coexistence [64] is not realistic in the present moment. To develop and promote a new paradigm of human–wildlife relations that resonates in ecosystems around the world, we require a common standard that helps people come to accept that their reality is intertwined with that of the animal other in the same way that a country’s constitution commands everyone’s loyalty [13]. In wildlife settings, the North American Model of Wildlife Conservation (NAM) catalyzes entente between segments of society about HWC, by, for example, supporting the lawful killing of wildlife for politically expedient (i.e., “legitimate”) reasons and means [65]. The NAM is rooted in an orthodoxy of permission tolerance and, thus, cannot help humanity envision radical potentials of human–wildlife interculturality.

Gradations of tolerating wildlife are necessary as they innately experiment to optimize their existence in a human-dominated world [54,66]. However, tolerance as the common standard to unify humans for wildlife must be temporary or provisional. Following Goethe [59] and Broder [67], tolerance should be a transitory attitude that leads to appreciation or a socio-cultural down payment toward grander ends, respectively. Societies need a new social–ecological contract from which to assign collective loyalty. Some might advocate undertaking a Habermasian reconstruction of tolerance [68] to rethink a coexistence paradigm; but, as I have tried to explain, any tolerance approach will be underscored by speciesism, negativity, violence, and short-term thinking. Hence, a reliance on the historical tolerance paradigm portends that we expect little change in human–wildlife relations and, thus, persistent HWC that does not actually produce a coexistence design necessary to sustain wildlife populations in the long term.

### 3.1. From Tolerance to Hospitality 

In search of an alternative standard for an increasingly divided world there has been a rise in scholarly interest in non-commodified hospitality over the last two decades [69]. Scholars have proposed that humans engage hospitality to espouse openness and welcoming to those unlike them. Though we do not always know what hospitality is and means in all situations, Derrida [70] explained that we tend to mean “bid someone welcome”, “accept”, “invite”, “greet”, or “receive” another to our home. Hospitality has been viewed as a civic duty because one has a right to hospitality [71], moral obligation [72], sensibility [73], principle [74], virtue [17], or restorative justice project [75], with intent to harmonize bonds among humanity, particularly between the more and less well-off [76]. In Kant’s [71] (p. 108) view, though not without disagreement, the pursuit of hospitality can one day yield shared “perpetual” peaceful relations and rejects hostility upon meeting the stranger, assuming the stranger does not behave aggressively or violently. Hospitality “must be expressed in goods and services offered without condition: food, shelter, care, acceptance, understanding, empathy, love, or welcome” [75] (p. 331). Though hospitality can be shaped by the conditions set upon it, what is shared by all sentient beings is Earth’s surface [71] and, thus, “mutual entanglement” [27] (p. 2), which theoretically provides rudimentary justification for the law of (unconditional) hospitality to be applied to everything everywhere and efforts to implement it.

The practice of hospitality is affected by a range of factors, which can lead to a sense of uncertainty about host–guest relations. As such, there are two forms of hospitality [70]. A conditional hospitality (position of terms and conditions) cannot be considered ethical as it hinges on the enforcement of and exclusions by the master from a home or nation, and on being denied sovereignty over self or home [77]. For this reason, conditional hospitality is argued to be inherently violent (e.g., incites unnatural decisions or actions [76]) and resembles a state of non-recognition inherent to tolerance. The host is hostile to those being hosted. Unconditional hospitality, on the other hand, would welcome any lifeform: “whether or not the new arrival is the citizen of another country, a human, animal, or divine creature, a living or dead thing, male or female” with no reservations or conditions, requiring an openness to the relationality of the temporality and the process [78] (p. 77). 

Versions of hospitality still struggle with anthropocentrism and hierarchical power structures [66,73,79,80]. Debating these criticisms and shortcomings is beyond the scope of this essay. However, these works are essential for problematizing and considering how to conceptualize what it means to be human [40] and the (il)logics and orientations that underpin current anthropocentric human–Nature relations in many parts of the globe [66]. Liberation from mastery and entitlement logics to create a human that cohabitates a multi-species planet [66] will not happen rapidly, nor will some societies feel it is necessary or applicable. Nonetheless, hospitality can help societies where, for example, private property or neoliberal logics are woven into the fabric of livelihoods, begin to see their “true ecology”, where Humanity is hosted by Nature. As articulated by Lind and Ferraro [40], a view which I espouse, such a project would “deanthropocentrize” hospitality because we would be required to conceive of and submit to a “true ecology” about the primordial human condition as interdependent on Nature and not separate from it. 

Thus, hospitality is a considerable upgrade from tolerance because (a) it does not comprise an objection component and embraces recognition of the other [81], (b) the grounds for objection are minimally disruptive and reasonable [20], and (c) humans are guests first and hosts second [40]. To Esteva [34] (p. 249), hospitality is the antithesis of tolerance: “To be hospitable is not to follow the other, to adopt their views, to affirm them, or to negate them” but to join roots and engage in a process of interculturality. (Indeed, animals have cultures, as I will explain below.) As guest (e.g., where suitable habitat for a non-human animal may be assigned host) or host (e.g., animal enters a human-dominated area), hospitality promotes a form of coexistence that requires humans to merge material and symbolic spheres that are uniquely designed to produce a sense of belonging for all species [81]. As guest or host, hospitality is a call to open mutualistic spaces and keep them open, engaging with the other by recognizing their specificities, a dynamic which turns host into guest and guest into host, thereby producing reverence for the other [73]. It is transactional and transcendent because one must be prepared to change their views, their relationships, and their way of being. For instance, it would require humans to wait for the raccoons who take up residence in an attic to leave on their own [82] or dismantle an indiscriminate killing or nuisance discourses when deer or predators take advantage or pass through human-altered landscapes [75,83]. It would also require depowering of, for example, wilderness and neoprotectionist discourses, which espouse separation of humans and Nature, allowing for humans to more freely see themselves as guests when occupying wildlife systems. 

Hospitality may seem utopian or abstract because it runs counter to the spirit of tolerance fundamentalism that many societies know so well. However, as tolerance emerged from an era of human suffering, hospitality can emerge from our willingness to pledge to being collectively and individually better off and dispose of the problematic hierarchical human–animal relations that have produced a degraded and inhospitable planet. Though few scholars have theorized hospitality towards Nature and non-human animals, the interest in the nexus of hospitality and human–wildlife interactions is emerging, often building upon Derrida’s deconstruction approach and appeal to assign moral status to animals. Derrida wrote of achieving a form of genuine hospitality that does not erase differences but preserves them to better consider the priority of animals [48]. To do so, Morton [84] argued that humans must hold sacred a law of non-contradiction which would not allow our species to claim progress where there is degradation and overcome our inability to reconcile the competing natures of Earth’s exploitation for human benefit and safeguarding ecological beings. Unlike tolerance, hospitality offers us the intellectual space required to rethink human–wildlife relations in a way that weakens and eventually reverses [40] the anthropocentric power dynamic undergirding tolerance and creates hospitable spaces for wild animals on a crowded planet. In the text that follows, I discuss five commitments humans can make to help hospitality become a social institution in global wildlife conservation.

### 3.2. A Path to Hospitality as a Social Institution

The first commitment is to engage ontological barriers that prevent reimagining of human–wildlife relations. Lind and Ferraro [40] proposed an ontological movement to construct a new self-knowledge of our interdependence on Nature promoting the idea that it hosts us, and we are the guests, though this relationship can be inverted in some contexts. This view suggests that hospitality in the context of HWC is as bound to logics of mutual relationalities as it is reciprocity [69]. Replacing thin or highly anthropocentric forms of coexistence with thick or mutualistic arrangements will be contested but not all ontological viewpoints about wildlife are valid or need to be accommodated [27]. Indeed, societies need to determine right and wrong or true and untrue, but these determinations can be conducted in an era of hospitality.

Reifying a new ontological perspective will require societal commitment to hospitality as a design principle in societal political thinking [13]. The benefits to ecosystems include but are not limited to clearly defined boundaries, monitoring, and sanctions to define and direct how thick coexistence is designed and facilitated. Over the last few decades, scholars have leaned into a Kantian [71] right to hospitality approach to establish a new social contract with Nature and achieve perpetual peace via diminished environmental degradation [85,86]. The payoff, as Cooke [87] advocated, is a “non-speciesist” rights-based hospitality that would yield enhanced freedom for wildlife to range and less violent interactions between humans and wildlife and, I add, between humans about wildlife. The prevalence of HWC and sheer number of researchers and practitioners espousing tolerance is evidence that we struggle to universally grant rights to wildlife due to ontological conflicts [73], and, thus, hospitable sentiments often do not apply to all wildlife such as snakes, rats, bats, or opossums. We are closer to achieving Kant’s [71] *right of visit* than we are to universal hospitality predicated on settlement rights or *right of residence*. Thus, we have work to do in this regard and can ask more of ourselves than awareness and appreciation for wildlife. As critical scholars argue, humans must make mutual respect, reverence, and dignity in all biodiversity as political values. Such a divergence from a charity-based permission form of tolerance may seem subtle, but it firmly reflects a shift in positive thinking where humans and animals both have a politically codified right to peacefully co-occupy the same space.

Because non-human animals cannot enter negotiations or communicate their needs verbally, models of functional coexistence between humans and wildlife need to be rethought. We can embrace a third commitment where we adapt human–human coexistence models to establish an MLSS that provides basic needs for wildlife with overlapping and competing needs. We can easily discern what wildlife has told us that they want and need through their behaviors, prospering, suffering, and decline. A framework outlining an MLSS borne of hospitality rather than tolerance can serve as a benchmark for long-term cultural and political change which resists and undermines structural violence and the inequity characteristic of functional coexistence [56]. An MLSS is needed to think broadly about the distinct nature of relationships affected by HWC and how the espousal of different forms of functional coexistence influences wildlife conservation outcomes and dynamics. Because humans can transform the earth on a mass scale [88] and HWC negatively affects wildlife in the short (e.g., disturbance) and long terms (e.g., evolutionarily, extinction, [89,90]), we need a fresh set of terms to govern how we interact with non-human animals and allow them to exist in their difference in more places (i.e., redefine MLSS). For example, decision makers can design policy attributes that promote hospitality (e.g., wildlife as beings) and reject those that promote negation (e.g., wildlife as things). Adapting Arai’s approach [56], we can unite functional coexistence and hospitality to conjure a policy attributes framework to identify underlying factors, such as issues, goals, identities, relationships, dynamics, and social structures, that perpetuate HWC through the denial of the right for wildlife to be who they are or exist in their own place:

- General categories of conflict actors;
- Reason for inhospitality (e.g., livestock eaten; systemic inequalities);
- Short-term approach to addressing intractable HWC conflicts (individual or collective: actions, project);
- Long-term approach to addressing intractable HWC conflicts (collective action: patterns, processes);
- Examples of factors/dynamics/practices that underpin non-hospitality or recognition of mutuality;
- Implications for governance (e.g., limits federal involvement; current land tenure system will remain unchanged).

Where negation occurs, normalizing grace rather than the impacts or damage (e.g., [91]), for example, can become a foundational element to design, administer, and assess HWC policy. Grace can be executed by yielding space, time, assistance, or honor [92]. 

Cultivating hospitality will need a commitment to dialogue and the creation of spaces for learning, experimentation, and truth seeking [17]. These aspects are requirements for societies to embrace more-than-human worlds [66], making space and time to understand the animal other. To overcome the problems with tolerance as a solution to HWC, we would do well to incorporate animal voice and agency within coexistence arrangements. The concept of sociality [93] can serve as a boundary object, an object whose meaning has sufficient commonality that all groups know though interpret differently, to better align with what is true and just for both humans and the animal other. The process requires a new science privileging human recognition of the animal other by establishing a dialogue of knowledges to moderate otherness with an understanding of animal sociality. Research suggests what a dialogue of animal cultures looks like if we merely spend more time trying to understand these cultures [94,95]. Technological advances provide unprecedented potential for humans to use sociality as a boundary object. For instance, researchers have been using artificial intelligence to construct interspecies communications, specifically between humans and non-human animals, cultivating a form of pattern identification through deep listening to improve understandings of animal cultures [96]. In turn, summoning Conway’s [17] wisdom, commitment to a science of sociality helps cultivate the amiable and respectful welcoming of animal cultures by uncovering the experience of wildlife in a language that both entities can negotiate to productive ends. We do need to help wildlife recognize and honor the ways of the host to make possible and sustain civil relations between humans and wildlife, and sociality should allow us to do so less violently. 

Finally, as societal commitment to tolerance as a collective moral duty to others has struggled [97], hospitality will require sufficient and organized motivations for universal adoption to become a social institution. It is popular to use anthropocentric, emotively charged metrics that privilege one’s satisfaction in human–animal exchanges or human acceptance or enduring of the animal other (the reader can examine psychometric studies using these scales for themselves). However, these measures do not force us to “decentre human positions of control” by becoming more vulnerable and opening cosmopolitical possibilities to consider the life of the animal other seriously because they do not attune and repose to known differences in a way that cultivates rather than tears apart mutuality [62] (p. 489). Our motivations to interact with the animal other requires the normalization of a social–ecological attuned cosmopolitics that embraces decentering and mutual accommodation in a way that represents a shared struggle to survive [27]. These motivations will need to be organized to produce hospitality at large scales and realize its collective potential. 

A process of organization will require multiple formal and informal channels and relations that recast hospitality as part of a large-scale reinforcing process of sincere welcoming, care, and support [68], and interdependence. Broadly, this normalization process will entail shifting responsibility to humans to modify their behavior to help keep wildlife invisible where it is ideal or essential, learning about what wildlife want and need, and ensuring they are not harmed for being themselves [27,98]. Though our obsession with wildlife obfuscates our need to confront our biases [65], practical solutions we can pursue today include adopting wildlife-friendly planning (e.g., wildlife corridors); altering our daily vocabulary [99]; rethinking the concept of home [100]; programs that mature human decision making [101]; depowering human deference to conditional hospitality and violence when refusing to normalize human–wildlife interactions that have been deemed problematic when they are not [27,65]; and governance designed to cultivate social and institutional value systems that are ecosystem- and wildlife-friendly over status quo arrangements moored to human mastery over Nature [54,102].

## 4. Conclusions

A tolerance paradigm has been a necessary step in cultivating human–wildlife relations, but the time for a new paradigm is upon humans. It has long been the belief of scholars that hospitality can improve human–human relations, and the time is right to explore this concept in the human–Nature and human–wildlife domains. In this paper, I outlined several common criticisms of tolerance as a conceptual and policy tool and how it produces dysfunctional coexistence arrangements between humans and wildlife. I proposed that an era of hospitality is required to mitigate growing human–wildlife conflict on a planet that is becoming increasingly crowded and degraded. Hospitality is a more practical and durable upgrade over tolerance but requires humans to commit to a novel social–ecological contract with each other, Nature, and non-human animals to effectively loosen tolerance’s grip on current systems of wildlife governance.

## Data Availability

No new data were created or analyzed in this study. Data sharing is not applicable to this article.
